# Supplementary material for: A Tgfbr1/Snai1-dependent developmental module at the core of vertebrate axial elongation
Source: eLife. 2020 Jun 29;9:e56615. doi: 10.7554/eLife.56615 (PMC7324159; doi:10.7554/eLife.56615)
Supplement: Transparent reporting form [file elife-56615-transrepform.pdf]

1st Floor
24 Hills Road
Cambridge CB2 1JP, UK

P 01223 855340
W elifesciences.org
T @elife

eLife's transparent reporting form

We encourage authors to provide detailed information *within their submission* to facilitate the interpretation and replication of experiments. Authors can upload supporting documentation to indicate the use of appropriate reporting guidelines for health-related research (see EQUATOR Network), life science research (see the BioSharing Information Resource), or the ARRIVE guidelines for reporting work involving animal research. Where applicable, authors should refer to any relevant reporting standards documents in this form.

If you have any questions, please consult our Journal Policies and/or contact us: editorial@elifesciences.org.

Sample-size estimation

- You should state whether an appropriate sample size was computed when the study was being designed
- You should state the statistical method of sample size computation and any required assumptions
- If no explicit power analysis was used, you should describe how you decided what sample (replicate) size (number) to use

Please outline where this information can be found within the submission (e.g., sections or figure legends), or explain why this information doesn't apply to your submission:

The number of cells analyzed in the single cell RNA-seq experiments was estimated using as a reference the numbers used in previously reported experiments, as our data had to be compared with the data from those published experiments.

The rest of the experiments involve expression data by in situ hybridization or immunofluorescence, for which each factor was tested in at least two embryos per genotype in independent experiments, consistently showing similar patterns with the exception of *Lfng*, which gave different patterns in the PSM region as reported in Figure 5 and Figure 5-figure supplement 1, and notochord markers in *Snai1-cKO* embryos, that gave two alternative patterns as specified in the main text.

Replicates

- You should report how often each experiment was performed
- You should include a definition of biological versus technical replication
- The data obtained should be provided and sufficient information should be provided to indicate the number of independent biological and/or technical replicates
- If you encountered any outliers, you should describe how these were handled
- Criteria for exclusion/inclusion of data should be clearly stated
- High-throughput sequence data should be uploaded before submission, with a private link for reviewers provided (these are available from both GEO and ArrayExpress)

Please outline where this information can be found within the submission (e.g., sections or figure legends), or explain why this information doesn't apply to your submission:

eLife Sciences Publications, Ltd is a limited liability non-profit non-stock corporation incorporated in the State of Delaware, USA, with company number 5030732, and is registered in the UK with company number FC030576 and branch number BR015634 at the address 1st Floor, 24 Hills Road, Cambridge CB2 1JP | August 2014

Expression of each gene or protein was tested by in situ hybridization or immunofluorescence in at least two embryos per genotype in independent experiments, consistently showing similar patterns.

Some variability was observed in *T-str-Snai1* transgenics as the described phenotype, caudal morphological abnormalities at E9.5, was observed in about half of the transgenic embryos. This is specified in the results section. Most likely reason for this variability is differences in transgene copy number.

High-throughput sequencing data used in this work was submitted to GEO and, as specified in the methods section, can be found in https://www.ncbi.nlm.nih.gov/geo/query/acc.cgi?acc=GSE147100 with the following accession number: GSE147100.

Statistical reporting

- Statistical analysis methods should be described and justified
- Raw data should be presented in figures whenever informative to do so (typically when N per group is less than 10)
- For each experiment, you should identify the statistical tests used, exact values of N, definitions of center, methods of multiple test correction, and dispersion and precision measures (e.g., mean, median, SD, SEM, confidence intervals; and, for the major substantive results, a measure of effect size (e.g., Pearson's r, Cohen's d)
- Report exact p-values wherever possible alongside the summary statistics and 95% confidence intervals. These should be reported for all key questions and not only when the p-value is less than 0.05.

Please outline where this information can be found within the submission (e.g., sections or figure legends), or explain why this information doesn't apply to your submission:

The p-values for Figures 1, Figure 1-figure supplement 1, Figure 2, Figure 2-figure supplement 1 and Figure 2-figure supplement 2 were calculated using the programs designed to evaluate single cell data as specified in the methods section. Exact p-values for each cluster gene marker are provided in the source data files, Figure 1- source data1 for Figure 1 and Figure 1-figure supplement 1, and Figure 2- source data1 for Figure 2, Figure 2-figure supplement 1 and Figure 2-figure supplement 2.

(For large datasets, or papers with a very large number of statistical tests, you may upload a single table file with tests, Ns, etc., with reference to sections in the manuscript.)

Group allocation

- Indicate how samples were allocated into experimental groups (in the case of clinical studies, please specify allocation to treatment method); if randomization was used, please also state if restricted randomization was applied
- Indicate if masking was used during group allocation, data collection and/or data analysis

Please outline where this information can be found within the submission (e.g., sections or figure legends), or explain why this information doesn't apply to your submission:

eLife Sciences Publications, Ltd is a limited liability non-profit non-stock corporation incorporated in the State of Delaware, USA, with company number 5030732, and is registered in the UK with company number FC030576 and branch number BR015634 at the address 1st Floor, 24 Hills Road, Cambridge CB2 1JP | August 2014

This information does not apply for our submission as the experiments were not prone to any kind of group allocation.

Additional data files ("source data")

- We encourage you to upload relevant additional data files, such as numerical data that are represented as a graph in a figure, or as a summary table
- Where provided, these should be in the most useful format, and they can be uploaded as "Source data" files linked to a main figure or table
- Include model definition files including the full list of parameters used
- Include code used for data analysis (e.g., R, MatLab)
- Avoid stating that data files are "available upon request"

Please indicate the figures or tables for which source data files have been provided:

For Figure 1 and Figure 1-figure supplement 1, source data was provided in Figure 1-source data1. For Figure 2, Figure 2-figure supplement 1 and Figure 2-figure supplement 2 source data was provided in Figure 2-source data1
